# Chronic orofacial pain and psychological distress: findings from a multidisciplinary university clinic

**DOI:** 10.22514/jofph.2025.057

**Published:** 2025-09-12

**Authors:** Parvaneh Badri, Ivonne Hernández, Justin Long, Maryam Amin, Reid Friesen

**Affiliations:** ^1^Oral Medicine, Mike Petryk School of Dentistry, Faculty of Medicine & Dentistry, University of Alberta, Edmonton, AB T6G 1C9, Canada; ^2^Mike Petryk School of Dentistry, Faculty of Medicine & Dentistry, University of Alberta, Edmonton, AB T6G 1C9, Canada

**Keywords:** Orofacial pain, Multidisciplinary clinic, Psychological distress, Temporomandibular joint (TMJ) pain, Chronic pain management

## Abstract

**Background**: Chronic orofacial pain (COFP) is a complex condition that requires multidisciplinary management grounded in the biopsychosocial model. This study examined the associations between temporomandibular disorders (TMD) and headache symptoms and psychological factors within a university-based multidisciplinary care setting, providing insight into the integration of mental health in COFP management. **Methods**: A retrospective review of 162 patient records from the University of Alberta Multidisciplinary Orofacial Pain Clinic (2020–2023) was conducted. Psychological assessments included the Adverse Childhood Experiences (ACE) scale, Pain Catastrophizing Scale (PCS) and Injustice Experience Questionnaire (IEQ). Logistic regression was used to evaluate associations between psychological factors and pain severity. **Results**: The cohort (aged 13–93) was predominantly female (84.0%). Fifteen percent declined psychological measures. Significant associations were observed between PCS (*p* = 0.036) and IEQ (*p* = 0.005) scores and reported pain severity. Moderate-to-high PCS scores were associated with a 3.67-fold increase in the odds of moderate to severe TMD symptoms (Odds Ratio (OR): 3.67, 95% Confidence Interval (CI): 1.09–12.35), while high PCS scores predicted severe headaches (OR: 3.91, 95% CI: 1.50–10.17, *p* = 0.005). Elevated IEQ scores were similarly associated with increased odds of severe headaches (OR: 2.76, 95% CI: 1.08–7.05, *p* = 0.034). **Conclusions**: Psychological factors such as pain catastrophizing and perceived injustice are strongly associated with symptom severity of TMD and headache symptoms in COFP. These findings underscore the importance of integrating targeted psychological assessments into multidisciplinary care. Further research should explore barriers to implementation and advance biopsychosocial approaches to improve outcomes for patients with COFP.

## 1. Introduction

Chronic pain significantly affects patients’ physical and emotional well-being and imposes a substantial financial burden on healthcare systems [1, 2]. In the United States, 25% of people reported having pain every day in the previous 3 months [3]. In Canada, approximately one in five individuals is estimated to experience chronic pain [4]. Chronic orofacial pain (COFP), which presents as ongoing discomfort in the face, mouth, head, or temporomandibular joint (TMJ), is commonly linked to conditions such as temporomandibular disorders (TMD), trigeminal neuralgia and various headache types [5]. The persistence and variability of COFP are influenced by psychological, social, genetic, and environmental factors, including stress and trauma highlighting the importance of a personalized, multidisciplinary approach to treatment [6].

The biopsychosocial model offers a comprehensive framework for understanding and managing COFP, emphasizing the integration of biological, psychological and social dimensions [7]. Pharmacologic interventions target underlying neurobiological mechanisms, while psychological therapies address maladaptive cognitive and emotional patterns contributing to chronic pain [8, 9]. Social interventions such as patient education and peer support also play a critical role [10, 11]. Multidisciplinary pain clinics exemplify this model, combining pharmacological, psychological, and social interventions to address the complexity of chronic pain. There is strong evidence that this approach improves pain severity, function, and psychological outcomes [12].

Psychological factors such as anxiety, depression, and stress are now recognized as critical modulators of pain perception and chronicity [13]. Recent research has shifted from general emotional distress to examining specific psychological constructs that influence chronic pain experiences. Tools such as the Adverse Childhood Experiences (ACE) survey [14, 15], the Pain Catastrophizing Scale (PCS) [16, 17, 18] and the Injustice Experience Questionnaire (IEQ) offer structured ways to identify vulnerabilities associated with pain amplification, persistence, and treatment resistance. The ACE survey captures the impact of early-life adversity on stress regulation and neurobiological sensitivity [14, 15]. The PCS measures maladaptive thought patterns like rumination, magnification and helplessness [17, 19]. The IEQ assesses perceived injustice, feelings of unfairness or blame, that may contribute to anger, intrusive thoughts, and reduced coping capacity [16].

The decision to include the ACE, PCS and IEQ instruments was guided by the biopsychosocial framework of chronic pain, with each tool selected for its relevance to psychological mechanisms implicated in pain persistence and disability. Specifically, the ACE captures early-life adversity, the PCS addresses maladaptive cognitive-emotional responses to pain, and the IEQ measures perceived injustice, a construct with growing empirical support in chronic pain literature. While screening tools such as the Patient Health Questionnaire-9 (PHQ-9), Generalized Anxiety-7 (GAD-7), and Beck Depression Inventory 2nd Edition (BDI-II) offer insight into broader emotional symptomatology and are standardized in other orofacial pain literature, our objective was to focus on psychological constructs with more direct relevance to pain modulation and functioning. Notably, recent evidence suggests that perceived injustice not only exacerbates pain-related interference but also contributes to the progression of depressive symptoms over time, independent of baseline affective status [20]. Its inclusion in the present study was intended to provide a deeper understanding of psychosocial risk factors unique to individuals with COFP, building upon insights gained from landmark studies related to the Diagnostic Criteria for Temporomandibular Disorders (DC/TMD) framework [21].

Although psychological distress has been widely studied in broader chronic pain populations, its specific impact on chronic orofacial pain subtypes, particularly TMD and chronic headache, remains underexplored [13]. While the PCS has been applied to general chronic pain cohorts, its use in orofacial multidisciplinary clinics is limited [17, 19]. Similarly, although the IEQ has been validated in musculoskeletal populations, it has rarely been applied to COFP cohorts [16]. Few studies have assessed these psychological constructs within real-world multidisciplinary care settings where psychological assessments are embedded into patient management pathways. Addressing this gap is critical to advancing biopsychosocial models of COFP care, in particular TMD and headache.

Accordingly, this study aimed to examine the associations between psychological factors, including ACE, PCS, and IEQ scores, and reported pain severity related to TMD and headaches in patients attending the University of Alberta’s orofacial multidisciplinary pain clinic (OMPC). By identifying these associations, we sought to provide insight into how integrated psychosocial assessments (Fig. 1), which illustrate the care model employed at OMPC, could inform personalized, patient-centered strategies within a biopsychosocial framework.

**Fig. 1.** School of dentistry outpatient chronic orofacial multidisciplinary pain clinic. TMJ: temporomandibular joint.

## 2. Material and methods

This retrospective chart review study involved the examination of consecutive patient records from the Outpatient Chronic Orofacial Multidisciplinary Pain Clinic, part of the Oral Medicine Graduate Program Clinic at the University of Alberta, Canada. Data were collected for the period between February 2020 and December 2023 to ensure a comprehensive sample while also accounting for disruptions resulting from the COVID-19 pandemic, which led to a six-month clinic closure from March to August 2020. Ethical approval was granted by the University of Alberta Research Ethics Board (Pro00112133_REN3). Due to the retrospective nature of the study, the requirement for participant consent was waived by the Ethics Board. The inclusion criteria encompassed all patients treated at the OMPC during the specified timeframe, with no exclusions applied. This study was conducted as part of a larger MSc thesis by Dr. Parvaneh Badri, titled “*Analysis of Referral Pathways, Diagnosis, and Treatment Patterns in a University-Based Orofacial Multidisciplinary Pain Clinic*”, completed at the University of Alberta, under the supervision of Dr. Reid Friesen and Dr. Maryam Amin.

The chart review was conducted using a structured ten-step methodology for retrospective medical record analysis, as described by Vassar and Matthew (Table 1) [22]. This approach allowed for systematic and consistent data extraction aligned with the study’s predefined objectives. Extracted variables included pre-existing mental health diagnoses, intensity ratings for jaw and headache pain, and psychological screening results based on three validated instruments: the Adverse Childhood Experiences (ACE) scale, the Pain Catastrophizing Scale (PCS) and the Injustice Experience Questionnaire (IEQ). To enhance data quality assurance, a random sample of 10 records was re-reviewed by the principal investigator to confirm accuracy and consistency in data entry. While a formal pilot was not undertaken, this verification step helped to support the reliability of the review process.

**Table 1.** Retrospective chart review steps.

Step Number	Methodological Component
1	Create well-defined, clearly articulated research questions
2	Consider sampling questions *a priori*
3	Operationalize variables included in retrospective chart review
4	Train and monitor data abstractors
5	Develop and use standardized data abstraction forms
6	Create a data abstraction procedure manual
7	Develop explicit inclusion and exclusion criteria
8	Address inter-rater and intra-rater reliability
9	Conduct a pilot test
10	Address confidentiality and ethical considerations

TMD diagnoses were established using the DC/TMD framework, applied by trained clinicians during initial assessment [21]. This ensured standardized classification within a multidisciplinary care context but other variables were not directly tied to the study’s analytic variables.

In this study, “jaw pain” referred to pain arising from either TMJ arthralgia or masticatory myalgia, as defined by the 2014 DC/TMD criteria. Although these conditions have different etiologies, both are musculoskeletal disorders of the masticatory system and present similarly, typically as pain in the jaw, temple, ear, or preauricular area, often worsened by jaw movement, function, or parafunctional habits such as clenching and grinding. Clinical findings commonly include familiar pain reproduced by palpation or jaw movement tests, including maximum opening and lateral excursions. Psychological and behavioral factors, such as stress and anxiety, may further exacerbate symptoms [23]. For this study, the two conditions were combined under the umbrella term “jaw pain” to reflect their overlapping clinical features and the functional impact experienced by patients. Jaw pain severity was assessed using a standard 0–10 Numeric Rating Scale (NRS), with 0 representing no pain and 10 the worst pain imaginable. Scores were categorized as no/mild (0–3), moderate (4–6), or severe (7–10), consistent with the thresholds used for chronic musculoskeletal pain [24].

In this study, the diagnosis of headaches encompassed primary headache types, predominantly tension-type headaches (TTH), with less than 20% classified as migraine headaches, both with and without aura. Although these headache types differ in pathophysiology, they frequently co-occur with chronic orofacial pain, particularly in patients with TMD. Multiple studies have found that both migraine and TTH are highly prevalent among TMD populations, with migraine present in over 40% and TTH in nearly 19% of cases [25, 26]. Headache pain severity was assessed using a standard 0–10 Numeric Rating Scale (NRS) completed at clinical intake, where 0 represented no pain and 10 represented the worst pain imaginable. Patients were grouped into three categories based on their reported headache pain and TMD NRS scores: no/mild (0–3), moderate (4–6) and severe (7–10), following conventions established in prior pain research. This aligns with established research, which categorizes pain intensity based on patient-reported severity using a numerical pain scale, where scores of 1–3 are mild, 4–6 are moderate, and 7–10 are severe [24, 27, 28].

The psychological and emotional dimensions of pain were assessed using three validated tools: the ACE survey, the PCS, and the IEQ. These tools were selected for their ability to provide a structured framework for evaluating key psychological factors that influence chronic pain experiences. Scoring of these three tests is detailed in Table 2. All available patient charts were incorporated into the descriptive analysis, with no exclusions applied.

**Table 2.** Psychological assessment score ranges.

No	Psychological Scales	Score Ranges	Score Categories
1	ACE: Adverse Childhood Experiences	Score: 0–10	● Low risk: Score of 0 ● Intermediate risk: Score of 1–3 without accompanying health conditions ● High risk: Score of 1–3 with health conditions or a score of 4 or higher
2	PCS: Pain Catastrophizing Scale	Score: 0–52	● Low risk: Score of 1–14 ● Moderate risk: Score of 15–25 ● High risk: Score of 26 or higher
3	IEQ: Injustice Experiencing	Score: 0–48	● Low risk: Score below 19 ● Moderate risk: Score of 19–29 ● High risk: Score of 30 or higher

### 2.1 Data collection

Patient charts were systematically reviewed to gather detailed data aligned with the study objectives. This process adhered to a standardized protocol utilizing structured forms, ensuring uniformity and dependability in data extraction. Two independent reviewers conducted the chart abstraction. Discrepancies between reviewers were resolved through consensus discussions to maintain the accuracy and integrity of the data. All patients assessed in the clinic during the study period were eligible for inclusion. Formal exclusion criteria were not applied for psychiatric comorbidities, cognitive impairments, or medical conditions. However, patients with incomplete psychological assessments or unclear diagnoses were excluded from the inferential analysis. Those who declined psychological screening were still included in descriptive analyses when demographic and clinical data were available.

### 2.2 Statistical analysis

Statistical analyses were conducted using SPSS version 29 (IBM, Armonk, NY, USA). Descriptive statistics were applied to summarize demographic and clinical data, with categorical variables expressed as frequencies and percentages, while continuous variables were reported as means with standard deviations.

Associations between psychological variables (ACE, PCS and IEQ scores) and response variables (jaw pain and headaches) were assessed using Pearson’s chi-square test. Fisher’s exact test was applied when expected cell counts were less than five. Statistical significance was defined as a *p*-value < 0.05. Given the modest sample size, we minimized the risk of overfitting by grouping predictor variables (ACE, PCS and IEQ) into low, moderate, and high-risk categories and limiting the number of predictors included in each logistic regression model. Although a formal power analysis was not conducted, findings, particularly those with marginal significance, should be interpreted with caution.

Logistic regression models were used to assess the relationship between psychological risk categories and NRS pain severity (moderate/severe *vs.* no/mild). Odds ratios (ORs) with 95% confidence intervals (CIs) were calculated to determine the likelihood of experiencing moderate to severe symptoms based on psychological scores. Pain severity was dichotomized into “no/mild” and “moderate/severe” categories for both jaw pain and headaches. Psychological factors (ACE, PCS and IEQ) were grouped into relevant risk levels based on validation and utilization of the questionnaire; with “low risk” serving as the reference category. Logistic regression models were limited to psychological predictors to maintain analytic focus and avoid model overfitting. Although variables such as age, gender, and socioeconomic factors were available in some patient records, they were not captured consistently across participants and exhibited substantial heterogeneity, precluding their reliable inclusion as covariates.

## 3. Results

### 3.1 Descriptive results

A total of 162 patient charts from the OMPC, covering the period from February 2020 to December 2023, were reviewed. Patient demographics, including age and gender, were presented in Table 3. Regarding mental health conditions, 40.1% of patients reported having two or more diagnoses, while 25.9% had no documented history of a mental health diagnosis. Anxiety was the most prevalent standalone mental health condition (20.4%), followed by insomnia (5.6%), post-traumatic stress disorder (PTSD) (4.3%), and depression (3.7%).

**Table 3.** Patient demographic and clinical characteristics.

Category	Variable	N (%)
Gender		
	Female	136 (84.0%)
	Male	26 (16.0%)
Age (yr)		
	Range	13–93
	Mean (SD)	46.7
Mental Health Disorders		
	Anxiety	33 (20.4%)
	PTSD	7 (4.3%)
	Depression	6 (3.7%)
	Insomnia	9 (5.6%)
	2 or more of above	65 (40.1%)
	No Mental Health Diagnosis	42 (25.9%)
Primary Chief Complaint		
	TMJ Dysfunction (limited movement, locking)	70 (43.2%)
	Jaw Pain	54 (33.3%)
	Sleep difficulties (including parafunction/bruxism)	28 (17.3%)
	Other (headaches, ear pain)	10 (6.3%)

*TMJ: temporomandibular joint; SD: standard deviation; PTSD: post-traumatic stress disorder.*

The most frequently reported primary chief complaint was TMJ dysfunction, including limited movement and locking, affecting 43.2% of the cohort. Jaw pain was the second most common complaint (33.3%), followed by sleep difficulties, such as bruxism or parafunction (17.3%). Other complaints, including headaches and ear pain, were reported by 6.3% of patients (Table 3).

Psychological assessment data indicated that 14.81% (n = 24) of participants chose not to complete the ACE, PCS and IEQ tools. Among those who responded, 67.90% were classified as high risk on the ACE, 25.93% on the PCS and 20.99% on the IEQ (Table 4).

**Table 4.** Pain severity (jaw pain and headaches) and psychological assessment scores.

Variable	Category	Frequency (n)	Percentage (%)
Jaw Pain			
	No Pain	17	10.49
	Mild	10	6.17
	Moderate	60	37.04
	Severe	75	46.30
	Total	162	100.00
Headache			
	No Pain	53	32.72
	Mild	9	5.56
	Moderate	40	24.69
	Severe	60	37.04
	Total	162	100.00
ACE Score			
	Decline to Answer	24	14.81
	Low Risk	28	17.28
	High Risk	110	67.91
	Total	162	100.00
PCS			
	Decline to Answer	24	14.81
	Low Risk	48	29.63
	Moderate Risk	48	29.63
	High Risk	42	25.93
	Total	162	100.00
IEQ			
	Decline to Answer	24	14.81
	Low Risk	61	37.65
	Moderate Risk	43	26.54
	High Risk	34	20.99
	Total	162	100.00

*ACE: Adverse Childhood Experiences; PCS: Pain Catastrophizing Scale; IEQ: Injustice Experience Questionnaire.*

For the final clinical diagnosis, jaw pain, including masticatory myalgia and/or TMJ arthralgia, was identified in 140 patients (86.4%). Degenerative changes were noted in 85 patients (52.5%), headaches were diagnosed in 50 patients (30.9%), and TMJ internal derangements were found in 60 patients (37.0%).

### 3.2 Inferential results

While chi-square tests found no significant associations for ACE scores and headache or jaw pain severity, chi-square tests did show significant associations for PCS and IEQ scores with headache pain severity. Specifically, moderate PCS scores were associated with approximately fourfold higher odds of experiencing moderate to severe TMD symptoms (OR: 3.67, 95% CI: 1.09–12.35, *p* = 0.036). High PCS scores were also significantly associated with severe headaches (OR: 3.91, 95% CI: 1.50–10.17, *p* = 0.005). Additionally, high IEQ scores were associated with a 2.76-fold increase in the likelihood of severe headaches (OR: 2.76, 95% CI: 1.08–7.05, *p* = 0.034) (Table 5). Although prior literature suggests that NRS pain categories may be influenced by catastrophizing tendencies, the mean PCS scores in our sample fell within the moderate range. These findings suggested that the applied severity thresholds were likely appropriate for our population and not significantly biased by high levels of catastrophizing [24].

**Table 5.** Logistic regression analysis of psychological factors and pain outcomes: comparing no/mild pain to moderate/severe pain.

Outcome	Explanatory	Score level	OR	95% CI	*p*-value
Jaw pain					
	ACE score	Low risk	1 (reference)		
		High risk	1.18	(0.40, 3.55)	0.763
	PCS score	Low risk	1 (reference)		
		Moderate	**3.67**	**(1.09, 12.35)**	0.036*
		High risk	1.22	(0.46, 3.27)	0.690
	IEQ score	Low risk	1 (reference)		
		Moderate	1.51	(0.52, 4.40)	0.450
		High risk	0.94	(0.33, 2.68)	0.915
Headache					
	ACE score	Low risk	1 (reference)		
		High risk	1.80	(0.74, 4.33)	0.192
	PCS score	Low risk	1 (reference)		
		Moderate	1.40	(0.62, 3.16)	0.411
		High risk	**3.91**	**(1.50, 10.17)**	0.005*
	IEQ score	Low risk	1 (reference)		
		Moderate	1.76	(0.78, 3.96)	0.174
		High risk	**2.76**	**(1.08, 7.05)**	0.034*

* and bold text indicate significant associations. This table shows the logistic regression analysis for psychological factors (ACE, PCS and IEQ scores) and their association with pain outcomes. Pain severity is categorized into two groups: no to mild pain vs. moderate to severe pain. The odds ratios (OR) represent the likelihood of experiencing moderate to severe pain compared to no to mild pain, based on psychological score levels (low, moderate and high risk). “Low risk” is the reference category for each psychological tool. ACE: Adverse Childhood Experiences; PCS: Pain Catastrophizing Scale; IEQ: Injustice Experience Questionnaire; OR: Odds ratios; CI: confidence intervals.

## 4. Discussion

The findings highlight the significant impact of psychological factors, such as PCS and IEQ, on COFP symptoms, underscoring the need for targeted psychological interventions in multidisciplinary pain management (Fig. 2).

**Fig. 2.** Biopsychosocial chronic orofacial multidisciplinary pain clinic flowchart. TMJ: temporomandibular joint.

The findings supported our hypothesis, revealing significant associations between COFP symptoms and psychological factors. Moderate and high PCS scores were associated with greater odds of severe jaw pain and headaches, while elevated IEQ scores were tied to severe headaches. Interestingly, while moderate PCS scores were significantly associated with jaw pain severity, high PCS scores were not, despite similar sample sizes in each group. One possible explanation for this discrepancy is the presence of unmeasured confounding factors, such as age, gender and socioeconomic factors, which were not included in the regression models. It is also possible that individuals with high PCS scores reflect a more complex or heterogeneous subgroup, where psychosocial influences interact differently with clinical presentation. The exclusion of potential confounders introduces a risk of residual confounding, and thus the observed association in the moderate PCS group should be interpreted with caution. Conversely, no significant correlations were identified between ACE scores and COFP symptoms, despite the notably higher prevalence of elevated ACE scores in this cohort (67.9%) compared to the general population (30%). This finding differs from previous research, which has associated elevated ACE scores with heightened pain intensity and greater emotional distress [29, 30, 31]. Although ACE scores have been associated with the development of chronic pain conditions in adulthood [32], limited research specifically addresses their relationship with COFP. This finding may reflect differences specific to the COFP cohort compared to other pain conditions, potentially influenced by factors such as resilience or social support. Additionally, the financial means required for patients to access our clinic may indicate a unique subset of individuals with greater resources, which could mitigate some of the impacts of early-life trauma. These distinctions highlight the need for further research to explore how such factors modulate the relationship between ACE scores and COFP symptoms. Moreover, the retrospective design limited our ability to examine the type and timing of ACEs in detail, which may have obscured more specific associations. Specific types of adverse experiences (*e.g.*, physical assault) may differentially impact COFP symptoms, but this level of granularity could not be captured in our retrospective data. For example, it may be the case that patients who survived physical assault as children may have more intense COFP symptoms, but this data was not compared on that granular level. Additionally, further data about client symptomatology may help clarify this part of the study. Data regarding PTSD incidence rates in people exposed to trauma content reports rates between 5.6% and 9.4%, indicating that this exposure may not be sufficient for developing trauma symptoms, but rather increase overall vulnerability [33, 34]. Further nuance and symptom sampling may clarify this facet of the data.

Similarly, high IEQ scores have been shown to negatively impact chronic pain management [35], with a systematic review associating IEQ with poorer outcomes in musculoskeletal pain conditions [36]. However, no studies to date have explored the relationship between IEQ and COFP within a multidisciplinary orofacial pain setting. This study is among the first to examine the IEQ in a chronic orofacial pain cohort, particularly within a multidisciplinary care setting. Our finding that elevated IEQ scores were associated with headache severity highlights its potential clinical relevance for screening and management. By contrast, the connection between PCS and COFP has been studied, though findings suggest that catastrophizing alone may not be a decisive factor in TMD outcomes [18].

Mental health conditions were prevalent among the clinic patients, with 74.1% of patients having at least one mental health diagnosis, and 40.1% having two or more of anxiety, depression, PTSD or insomnia. Given the retrospective nature of this study, these diagnoses were derived from chart review rather than standardized psychiatric interviews, which may underestimate the true prevalence. These conditions are known to exacerbate pain perception and complicate treatment strategies, aligning with prior research [37, 38]. The high prevalence of anxiety and depression, in particular, underscores the need for integrated psychological interventions. Strategies such as cognitive-behavioral therapy are considered to be the gold standard in treating these features and further inclusion of techniques from this modality may be particularly effective in addressing these comorbidities [39]. The overlap between COFP and mental health disorders underscores the importance of treatment strategies that target both physical pain and psychological well-being. Therefore, implementing a multidisciplinary approach that combines physical, psychological, and social aspects of care is essential for improving patient outcomes [38]. For example, novel treatment/assessment paradigms such as the “Life Stress EAET Model” [40] may assist clinicians in gathering historical data while reducing patient stress symptoms. Emerging literature highlights the role of structural and physiological factors, such as sleep-disordered breathing, cervical posture, vagal nerve involvement and craniofacial morphology, in modulating both pain and psychological symptoms [41, 42]. Features like retrognathia, narrow dental arches, Class II malocclusion, and vertical growth patterns may reduce airway space and impair sleep, contributing to altered pain processing and neurotransmitter imbalances, including gamma-aminobutyric ccid (GABA)-related mechanisms [43]. Although these variables were not captured in our dataset, we acknowledge their clinical relevance and recommend that future studies incorporate airway and structural assessments alongside psychological screening to better understand the multifactorial nature of chronic orofacial pain.

To enhance statistical robustness and maintain analytic stability, we dichotomized pain severity scores for both jaw and headache symptoms into “no/mild” and “moderate/severe” categories. This decision was methodologically grounded in the context of our retrospective design and modest sample size, where overly granular categorization would have increased the risk of model overfitting and reduced the power to detect meaningful associations. In clinical pain research, particularly in studies involving patient-reported outcomes such as the Numeric Rating Scale (NRS), dichotomization is frequently used to facilitate interpretability, simplify model structure, and highlight clinically relevant thresholds of symptom severity. The thresholds applied in this study are consistent with previously validated cutoffs in chronic musculoskeletal pain populations [24].

Many of this study’s limitations stem from its retrospective design. Psychological questionnaire data were missing for 14.8% of participants, potentially impacting our findings. Patients cited non-completion reasons such as mistrust in the healthcare system, stigma surrounding psychological conditions and anxiety related to the COVID-19 pandemic, which also led to a six-month clinic closure. Financial barriers related to the fee-for-service model may have further limited access for some patients, introducing selection bias. Due to inconsistent documentation, we were unable to apply formal exclusion criteria for important confounding factors such as severe psychiatric disorders, cognitive impairments, substance use, and systemic medical conditions. Similarly, variables such as age, sex, sociodemographic characteristics, and prior mental health conditions were not consistently recorded and thus were not incorporated into regression models. Although the sample size and event rates would have supported additional predictors, we deliberately restricted the analysis to psychological variables. Important potential confounders were not included because the underlying data were incomplete and highly heterogeneous. As a result, the possibility of residual confounding cannot be excluded and should be considered when interpreting the study findings. In addition, our reliance on self-reported measures introduces potential reporting bias, and the generalizability of the results may be limited to multidisciplinary, university-based clinic populations. While dichotomization can reduce statistical power and obscure subtle gradations in symptom intensity, in our context it provided a streamlined analytic framework that enabled robust detection of significant associations between psychological constructs and pain severity [44, 45]. This approach was particularly important given the exploratory nature of the study and the need to balance statistical rigor with practical constraints inherent in real-world clinical datasets. Nonetheless, we recognize that this analytic choice may have attenuated sensitivity to specific patient subgroups, particularly those experiencing overlapping TMD and headache symptoms, and may have contributed to the absence of significant findings in some comparisons. Although widely used tools such as the PHQ-9 and GAD-7 were not included, our focus on the ACE, PCS, and IEQ reflected a targeted effort to examine psychological constructs directly relevant to chronic orofacial pain mechanisms. Despite these limitations, the findings underscore the critical role of psychological factors in jaw pain and headache management. The observed associations between PCS and IEQ scores and pain severity suggest that multidisciplinary clinics may benefit from routine psychological screening to identify patients at higher risk for severe symptoms. Incorporating these tools into clinical intake could support targeted referrals to therapies such as cognitive-behavioral therapy, while also enabling tailored education, improved communication about prognosis and strategies to enhance adherence. This integrated approach may be especially valuable for patients with high levels of perceived injustice or maladaptive pain beliefs, and supports the broader implementation of biopsychosocial care models in COFP treatment.

Future research should investigate how psychological factors, including ACE, PCS and IEQ, contribute to the persistence and progression of chronic orofacial pain over time. Longitudinal designs with larger, more diverse samples would allow for more comprehensive modeling of how these constructs interact with sociodemographic, behavioral, and structural factors. In particular, future studies should incorporate assessments of airway patency, sleep quality, Mallampati score, vagal tone, and craniofacial and cervical posture, as these variables may influence pain modulation through autonomic and neurochemical mechanisms. Anatomical features such as retrognathia, narrow dental arches, and vertical growth patterns have been associated with reduced airway space, which may contribute to alterations in neurotransmitter systems such as GABA, with downstream effects on both pain and psychological symptoms. Evaluating the contribution of these physical and physiological variables alongside validated psychological measures would help clarify the complex interplay between somatic and emotional dimensions of chronic orofacial pain. Further, research into the effectiveness of targeted interventions addressing cognitive and affective processes, including perceived injustice, remains a promising direction. Attention should also be given to reducing barriers that limit engagement with psychological screening and care, including stigma, mistrust, and lack of access. Finally, prospective designs should include standardized collection of demographic, psychological, and clinical variables to support more refined modeling and control of potential confounders.

## 5. Conclusions

This study underscores the importance of psychological factors, such as PCS and IEQ scores, in COFP, particularly their association with severe jaw pain and headaches. Integrating psychological assessments into multidisciplinary management is crucial to address the cognitive and emotional aspects of COFP. Although no significant association was identified between ACE scores and COFP symptoms, the notably high prevalence of elevated ACE scores emphasizes the need for further investigation into the impact of early-life trauma on various chronic pain conditions. Future research should aim to expand the biopsychosocial understanding of COFP and inform the creation of comprehensive, patient-centered treatment strategies. As chronic orofacial pain continues to challenge traditional care models, studies like this one underscore the urgency of integrating psychological assessment and support into front-line dental and medical care.
